# Blood–Brain Barrier (BBB)-Permeable Ethanolamine-Type Plasmalogens 18:0/20:4 (PE-PLS 18:0/20:4) Ameliorate Cognitive Impairments in Aged Mice

**DOI:** 10.3390/nu18193179

**Published:** 2026-09-26

**Authors:** Jiale Yu, Yiran Zhang, Ziyu Qiao, Hanyao Liu, Wen Zhang, Xintong Wang, Wei Xiong, Bing Fang, Fazheng Ren, Yanan Sun, Yixuan Li

**Affiliations:** 1 Key Laboratory of Precision Nutrition and Food Quality, Department of Nutrition and Health, China Agricultural University, Beijing 100193, China; yu15736219404@163.com (J.Y.); yiran1119@cau.edu.cn (Y.Z.); ziyyqiao@cau.edu.cn (Z.Q.); hanyaoliu@cau.edu.cn (H.L.); zwen@cau.edu.cn (W.Z.); xtwang@cau.edu.cn (X.W.); bingfang@cau.edu.cn (B.F.); renfazheng@263.net (F.R.); 2 Food Laboratory of Zhongyuan, Luohe 462300, China; xiongwei910702@126.com

**Keywords:** plasmalogens, cognitive impairment, synaptic plasticity, BDNF/TrkB pathway

## Abstract

**Background**: Plasmalogens (PLS) are a distinctive class of glycerophospholipids with reported neuroprotective properties; however, the mechanisms underlying their potential benefits for age-related cognitive decline remain incompletely understood. **Methods**: A D-galactose-induced mouse model of aging was used to evaluate the effects of PLS on cognitive performance. Hippocampal synaptic remodeling was assessed by examining the expression of synaptic proteins and receptors. An in vitro BBB model was used to evaluate the transport of PE-PLS 18:0/20:4. Molecular interaction analyses were conducted to investigate the potential binding of PE-PLS 18:0/20:4 to the brain-derived neurotrophic factor (BDNF) and its association with BDNF/TrkB signaling activation. **Results**: High-dose PLS supplementation significantly improved spatial memory and novel object recognition in D-galactose-treated mice. Molecular interaction analysis indicated that PE-PLS 18:0/20:4 could interact with BDNF through multiple hydrogen bonds. This interaction was associated with activation of the BDNF/TrkB signaling pathway, increased synaptic protein expression, and neuroprotective effects. **Conclusions**: PLS may mitigate age-related cognitive dysfunction by promoting hippocampal synaptic remodeling. PE-PLS 18:0/20:4 may contribute to these effects through BBB transport and modulation of BDNF/TrkB signaling, supporting the potential of plasmalogens as a nutritional strategy for promoting healthy brain aging.

## 1. Introduction

The global trend of population aging is irreversible, elevating age-related health challenges to a critical public health priority [1]. Due to its relatively limited antioxidant defenses, the brain is often among the earliest organs affected by aging [2]. Brain aging represents a key driver of cognitive disorders such as Alzheimer’s disease [3]. The underlying pathological mechanisms are multifactorial, encompassing accumulated oxidative stress, mitochondrial dysfunction, and progressive synaptic impairment—all of which collectively undermine autonomy and quality of life in the elderly [4,5,6].

Synapses, the fundamental units of neuronal communication, are central to learning and memory [7]. Their structural and functional integrity relies on the coordinated activity of presynaptic proteins, postsynaptic proteins, and neurotransmitter receptors [8,9,10]. However, aging-related oxidative stress and neuroinflammation contribute to reduced dendritic spine density, downregulation of key synaptic proteins, and ultimately cognitive decline [11,12,13]. Although several interventions—including the N-methyl-D-aspartate (NMDA) receptor antagonist memantine, the calcium channel modulator gabapentin, and certain neurotrophic factors—have been investigated for synaptic protection, their clinical application has been limited by suboptimal efficacy, significant side effects, or poor BBB permeability [14,15,16]. Thus, identifying effective strategies to preserve synaptic structure and function remains an important research priority.

Plasmalogens (PLS) represent a unique class of glycerophospholipids distinguished by a vinyl-ether bond at the sn-1 position, which endows them with distinct biophysical and biochemical properties [17]. They are highly enriched in neuronal membranes and myelin sheaths, where they contribute to membrane organization, antioxidant defense, and the maintenance of neural function [18,19,20]. Increasing evidence has therefore linked reduced PLS levels to aging and age-associated neurological disorders, including Alzheimer’s disease [21,22,23]. Marine-derived PLS have attracted particular interest as dietary bioactive lipids. As summarized by Yamashita et al. [24], marine PLS have been associated with the regulation of oxidative stress and inflammation, and both animal and human studies suggest potential benefits for age-related cognitive impairment. These findings support the potential of marine PLS as nutritional components for maintaining brain health during aging. However, most previous studies have evaluated PLS as lipid mixtures or at the lipid-class level, leaving the biological roles of individual molecular species insufficiently understood. In particular, it remains unclear which specific PLS species contribute to neuroprotective activity, whether exogenous PLS can cross the blood–brain barrier (BBB), and through which molecular targets and signaling pathways they exert their effects [25,26].

This study evaluated the effects of scallop-derived PLS on cognitive function in a D-galactose-induced aging mouse model and explored the underlying mechanisms related to synaptic plasticity. We further characterized specific PLS molecular species, assessed their BBB permeability, and investigated the interaction between PE-PLS 18:0/20:4 and BDNF. The findings provide novel experimental insights into the neurobiological mechanisms of PLS and support its potential as a nutritional intervention for promoting cognitive health in aging populations.

## 2. Materials and Methods

### 2.1. Animal and Experimental Design

We extracted PLS from scallops. The purity of the scallop-derived PLS extract was confirmed to be 96.78% [26]. Six-week-old female Kunming (KM) mice (SPF Biotechnology Co., Ltd., Beijing, China) were housed at the Animal Experiment Center of China Agricultural University under controlled conditions (12 h light/dark cycle; 22 ± 1 °C) and acclimatized for 7 days before the experiment. Animals were monitored daily for body weight, physical condition, activity, and signs of pain or distress. Humane endpoints were predefined, and mice showing >15% body weight loss or severe deterioration in general condition were to be euthanized immediately. No adverse events requiring premature euthanasia occurred. Mice were randomly assigned to four groups (*n* = 6/group). The control group received 2% Tween-80 orally and 0.9% saline subcutaneously; the model group received 2% Tween-80 and D-gal (400 mg/kg/day); and the PLS L and PLS H groups received PLS at 0.09 mg/kg/day and 0.3 mg/kg/day, respectively, together with D-gal. All procedures were approved by the Animal Welfare and Animal Experiment Ethics Review Committee of China Agricultural University (Approval No. AW60806202-5-04).

### 2.2. Y-Maze Test

The Y-maze test assessed short-term working and spatial reference memory in mice. The procedure comprised training and test phases. During training, mice were placed in the start arm (Arm A) with the novel arm (Arm C) blocked, allowing 15 min of free exploration between the start and familiar arm (Arm B) to establish spatial memory. After a 1 h interval, the test phase began: the novel arm was opened, and mice were returned to the start arm for 5 min of free exploration across all three arms. Arm entry sequences were recorded using a computerized tracking system (Shanghai Xinruan Technology, Shanghai, China). A spontaneous alternation was defined as consecutive entries into three different arms (e.g., ABC, BCA). The alternation rate was calculated as: (Number of spontaneous alternations/[Total arm entries − 2]) × 100%.

### 2.3. Tissue Collection

Mice were deeply anesthetized with sodium pentobarbital before transcardial perfusion (50 mg/kg, intraperitoneally). One hemisphere was dissected on ice to isolate the hippocampus and cerebral cortex. The tissues were immediately snap-frozen in liquid nitrogen and stored at −80 °C for subsequent molecular analyses. Of the contralateral hemisphere, one portion was immersed in Golgi–Cox impregnation solution for Golgi staining, whereas the remaining portion was fixed in freshly prepared 4% paraformaldehyde at 4 °C for subsequent paraffin embedding.

### 2.4. Hematoxylin and Eosin (H&E) Staining

Tissue sections were processed using a commercial H&E staining kit (G1120, Solarbio, Beijing, China). The sections were mounted using neutral resin for microscopic examination.

### 2.5. Golgi Staining

Brain tissues were trimmed into 2–3 mm blocks and promptly immersed in Golgi-Cox staining solution (GP1152, Servicebio, Wuhan, China) for 7 days at 4 in the dark. Subsequently, 100–150 μm sections were prepared and incubated in ammonia solution.for 60 min in darkness. After rinsing with distilled water, sections were incubated in sodium thiosulfate solution until turning brownish-black, rinsed again, and dehydrated through a graded ethanol series (70%, 80%, 95%, 100%). Finally, sections were cleared in xylene, mounted, and imaged for neuronal morphology analysis using ImageJ 1.53 (National Institutes of Health, Bethesda, MD, USA).

### 2.6. Measurement of the Hippocampal Neurotransmitters

Acetylcholine (ACh), 5-Hydroxytryptamine (5-HT), γ-Aminobutyric acid (GABA), and norepinephrine (NE) levels in the hippocampus were measured using enzyme-linked immunosorbent assay (ELISA) kits (A105-2-1&H104-1-1&H168-1-1, Jiangcheng Bioengineering, Nanjing, China) (E-EL-0047, Elabscience, Wuhan, China).

### 2.7. Western Blot

Tissues were lysed using RIPA buffer (Beyotime, Shanghai, China) for 30 min on ice and then centrifuged at 12,000× *g* for 15 min at 4 °C to obtain the total protein. Primary antibodies, including anti-α-Tubulin, anti-Synaptophysin (SYP) (66031-1&17785-1, Proteintech, Wuhan, China); anti-N-methyl-D-aspartate receptor subunit 1 (GluN1), anti-N-methyl-D-aspartate receptor subunit 2A (GluN2A) (A7167&A19089, Abbkine, Wuhan, China); anti-α-amino-3-hydroxy-5-methyl-4-isoxazolepropionic acid receptor (AMPAR), anti-γ-aminobutyric acid receptor (GABAR), anti-Postsynaptic density protein-95 (PSD-95), anti-Brain-derived neurotrophic factor (BDNF) (ab183797&ab55051&ab238135& ab108319, Abcam, Cambridge, UK); and anti-Tropomyosin receptor kinase B (TrkB) (4621T, CST, Danvers, MA, USA).

### 2.8. Quantification of PLS by Ultra Performance Liquid Chromatography-Tandem Mass Spectrometry (UPLC-MS/MS)

For solid samples, 10 mg of each was weighed into a 1.5 mL Eppendorf tube containing 10 stainless steel grinding beads and 20 μL of ultrapure water. Samples were homogenized for 3 min, followed by the addition of 120 μL of methanol and an additional 3 min of homogenization. After centrifugation at 18,000× *g* for 20 min at 4 °C, 10 μL of the supernatant was transferred to a fresh tube and mixed with 30 μL of internal standard-containing lipid extraction solvent. Following vortexing for 10 min and another centrifugation (18,000× *g*, 10 min), 20 μL of the supernatant was combined with 80 μL of lipid dilution solvent and transferred to an autosampler vial for LC-MS analysis.

Serum samples (10 μL) were transferred to a 96-well plate, mixed with 300 μL of methanol containing 5 mM ammonium acetate, vortexed for 20 min, and centrifuged (4000× *g*, 20 min, 4 °C). Then, 20 μL of the supernatant was diluted with 80 μL of methanol (5 mM ammonium acetate) for subsequent analysis.

For HBSS perfusate, 500 μL of sample was mixed with 500 μL of chloroform containing internal standards in a 96-well plate. After 20 min of vortexing and centrifugation (4000× *g*, 20 min), 450 μL of the lower organic phase was collected, dried under nitrogen, and reconstituted in 100 μL of methanol (5 mM ammonium acetate) for LC-MS analysis.

Quantification strategy. For serum and hippocampal samples, an untargeted lipidomics approach with internal standard-based semi-quantification was employed to compare the relative abundance of individual plasmalogen species because it was not possible to obtain authentic standards for all detected species. The instrument settings are provided in Appendix A Table A1, and QC reproducibility data for hippocampal tissue are provided in Supplementary Table S3. For the in vitro blood–brain barrier permeability assay, the screened candidate PE-PLS 18:0/20:4 was absolutely quantified using an authentic standard and a calibration curve. The MRM parameters for PE-PLS 18:0/20:4 are provided in Supplementary Table S1, and the authentic standard and calibration curve information is provided in Supplementary Table S2. Representative MRM chromatograms for the QC sample, authentic standard, and the three experimental groups are shown in Supplementary Figure S1.

### 2.9. Cell Culture Procedures

HT-22 cells and bEnd.3 cells were seeded into 10 cm dishes (DMEM with 10% FBS and 1% penicillin-streptomycin), then incubated at 37 °C with 5% CO_2_ for the subsequent experiments.

### 2.10. In Vitro the Blood–Brain Barrier (BBB) Integrity Assessment

#### 2.10.1. Transepithelial Electrical Resistance (TEER) Measurement

bEnd.3 cells were seeded into Transwell inserts (12-well, 0.4 μm pore size, Corning, New York, NY, USA) at a density of 3 × 10^5^ cells/well. TEER was recorded every other day using a Millicell ERS-2 Voltohmmeter (EMD Millipore, Burlington, MA, USA) to monitor monolayer integrity. Prior to measurement, electrodes were disinfected in 70% ethanol and equilibrated in pre-warmed HBSS. TEER values were calculated as: TEER (Ω·cm^2^) = (R_sample − R_blank) × 1.13, where 1.13 cm^2^ represents the surface area of the Transwell membrane.

#### 2.10.2. Four-Hour Hydrostatic Permeability Test

To evaluate the barrier’s mechanical integrity, a >0.5 cm liquid height difference was created between the apical and basolateral chambers. The difference was remeasured after 4 h of incubation at 37 °C. A persistent liquid level difference indicated intact barrier function.

### 2.11. In Vitro Evaluation of PLS Trans-BBB Permeability

To assess the ability of PLS to cross the BBB model, 0.5 mL of PLS-containing HBSS was added to the apical chamber, and 1.5 mL of blank HBSS to the basolateral chamber. The Transwell plates were incubated at 37 °C and 50 rpm for 4 h. Media from both chambers were used for PLS quantification.

### 2.12. Cell Viability Assay (CCK-8)

HT-22 (5 × 10^3^ cells/well) and bEnd.3 (1 × 10^4^ cells/well) cells were seeded in 96-well plates. Cells were treated with varying concentrations of D-gal (0–0.4 M) for 24 h. Cells were further treated with a concentration gradient of PLS (0–100 μg/mL) for 24 h (HT-22) or 4 h (bEnd.3) with a determined concentration of D-gal. After treatment, the medium was replaced with 100 μL of serum-free medium containing 10% CCK-8 reagent (CK04, Dojindo, Kumamoto, Japan) and then incubated at 37 °C for 30 min in the dark. Absorbance at 450 nm was measured, and cell viability (%) was calculated as:

[(OD_sample − OD_blank)/(OD_control − OD_blank)] × 100%

### 2.13. Molecular Docking

The three-dimensional coordinates for BDNF (PDB ID: 1BND) were retrieved from the Protein Data Bank and prepared by removing water, ions, and ligands using PyMOL 2.6.0a0 (Schrödinger, LLC, New York, NY, USA). Polar hydrogens and Kollman charges were added in AutoDockTools 1.5.6 (Molecular Graphics Laboratory, The Scripps Research Institute, La Jolla, CA, USA), and the receptor was saved in .pdbqt format. Ligand structures were downloaded from PubChem and converted to .pdbqt using Open Babel 2.4.0. Docking grid boxes were defined to cover the active sites, and docking was performed using AutoDock Vina 1.2.6 (Molecular Graphics Laboratory, The Scripps Research Institute, La Jolla, CA, USA). Binding poses were visualized with PyMOL.

### 2.14. Cellular Thermal Shift Assay (CETSA)

CETSA was employed to assess PLS binding to target proteins through thermal stabilization. A 10 mg/mL stock solution of PLS was prepared by drying the chloroform-dissolved standard under nitrogen and reconstituting it in DMSO. The optimal working concentration was determined via CCK-8 assay. HT-22 cells at ~80% confluency were treated with PLS or DMSO (vehicle) for 24 h, harvested, washed with PBS containing protease inhibitors, and lysed through three freeze–thaw cycles in liquid nitrogen. Lysates were aliquoted (100 μL) and heated at six temperatures (40–65 °C) for 3 min in a PCR thermal cycler. After centrifugation (20,000× *g*, 20 min, 4 °C), supernatants were collected for Western blot. The remaining soluble protein was used to construct thermal denaturation curves and evaluate PLS-induced changes in protein stability.

## 3. Results

### 3.1. PLS Attenuated Neuronal Damage in Mice with Cognitive Impairment

During the intervention period, PLS administration did not result in any significant differences in general physiological parameters across the groups (*p* > 0.05), indicating that neither the PLS treatment nor the D-galactose-induced aging model adversely affected the overall health status of the mice (Figure 1A). In the Y-maze test, spontaneous alternation rate serves as an indicator of spatial memory. Compared with the control group, the model group showed a significant decrease in this rate by 18% (*p* < 0.05). In contrast, high-dose PLS treatment increased the alternation rate by 26% (*p* < 0.05), restoring it to a level comparable with the control group (Figure 1B,C).

The number and structural integrity of healthy neurons constitute the biological basis for cognitive function, and cognitive impairment is closely associated with progressive neuronal loss and morphological abnormalities. In this study, neuronal cell bodies in the Cornu Ammonis area 1 (CA1), Dentate Gyrus (DG), and Cornu Ammonis area 3 (CA3) regions of the hippocampus were examined under light microscopy. Compared with the control group, neurons in the model group exhibited clear signs of degeneration, including nuclear pyknosis, hyperchromasia and necrosis. Notably, high-dose PLS treatment significantly reduced the number of neuronal lesions in the DG region (*p* < 0.05; Figure 1D), whereas no significant changes were observed in cortical regions (Figure 1E).

### 3.2. PLS Ameliorated Synaptic Structural Damage in Mice with Cognitive Impairment

Dendritic spines, as small membranous protrusions on neuronal dendrites, are key structural sites for memory formation and storage in the brain, while dendritic length and branching constitute critical morphological indicators of synaptic integrity. Golgi staining revealed that, compared to the control group, mice in the model group displayed a 60% reduction in dendritic spine density in the hippocampal DG region, accompanied by significant decreases of 33% in dendritic length and 47% in dendritic branching (*p* < 0.001). Furthermore, the dendritic organization appeared disorganized, suggesting that D galactose administration induced structural disruption in neuronal networks. In contrast, high-dose PLS treatment significantly increased dendritic spine density by 144% and improved dendritic length and branching by 42% and 26%, respectively (*p* < 0.001, *p* < 0.001, *p* < 0.05), while promoting a more orderly dendritic arrangement compared to the model group (Figure 2A–E).

### 3.3. PLS Ameliorated Synaptic Dysfunction in Mice with Cognitive Impairment

To assess the effects of PLS on synaptic function, we evaluated hippocampal neurotransmitter levels and synaptic receptor expression. Compared with the control group, the model group exhibited significantly reduced expression of GABAR, AMPAR, GluN2A, and GluN1 in the hippocampus, with decreases of 48%, 80%, 67%, and 67%, respectively (*p* < 0.05, *p* < 0.05, *p* < 0.001, *p* < 0.05), indicative of synaptic dysfunction. High dose PLS treatment significantly restored the expression of GluN2A by 143%, AMPAR by 270%, and GABAR by 89% (*p* < 0.05, *p* < 0.001, *p* < 0.05), whereas GluN1 expression showed an increasing trend without reaching statistical significance (*p* > 0.05) (Figure 3A–E). Consistent with these protein level changes, mRNA expression of the four genes followed a similar pattern (Figure 3F–I).

Cognitive impairment is closely linked to dysregulation of the neurotransmitter system. Our results demonstrated that high dose PLS treatment significantly elevated hippocampal levels of 5 HT, NE, and GABA by 16%, 80%, and 68%, respectively, compared with the model group (*p* < 0.05, *p* < 0.05, *p* < 0.001) (Figure 3J–L). In contrast, no significant differences in Ach levels were observed among the groups (Figure 3M).

### 3.4. Oral Administration of PLS Restored Plasmalogen Levels in the Blood and Brain of Mice with Cognitive Impairment

In previous work, the purity of the scallop-derived plasmalogen (PLS) extract was confirmed to be 97% [26]. However, the crude scallop extract contains a variety of phospholipid species. To characterize the specific plasmalogen profile in vivo, we analyzed the distribution of different PLS species in the serum and hippocampus of mice following oral administration. A total of 68 plasmalogen species were identified in the gavage samples. Subsequent analysis focused on the 20 most abundant species, which were selected by ranking all detected species according to their relative abundance and collectively accounted for more than 90% of the total plasmalogen content. These 20 species comprised 15 ethanolamine-type plasmalogens (PE PLS) and 5 choline-type plasmalogens (PC PLS). At the sn 1 position, fatty acyl chains were predominantly C18:0 (stearic acid), C16:0 (palmitic acid), and C18:1 (oleic acid), whereas the sn 2 position was enriched with polyunsaturated fatty acids (PUFAs), notably arachidonic acid (AA) and docosahexaenoic acid (DHA) (Figure 4A).

Following oral administration of PLS, the total plasmalogen content in the serum of model mice decreased by 16% (19.71 ± 0.83 μmol/L) compared with the control group, whereas PLS treatment restored the level by 13% (22.28 ± 1.03 μmol/L) relative to the model group (Figure 4B). Similarly, hippocampal plasmalogen levels were significantly reduced by 43% in model mice (*p* < 0.05) and markedly increased by 63% after PLS intervention (*p* < 0.05) (Figure 4C). In the hippocampus, 53 plasmalogen species were elevated following PLS treatment. Focusing on the 20 most abundant species, 18 were PE PLS and 2 were PC PLS (Figure 4D). Notably, PE PLS 18:0/20:4, PE PLS 18:0/22:6, and PE PLS 18:1/22:6 showed the most pronounced upregulation, consistent with the major plasmalogen species detected in the gavage samples (Figure 4D). These findings suggest that orally administered PLS can cross the blood–brain barrier (BBB) and exert functional effects in the brain.

### 3.5. PE-PLS Was Able to Penetrate the BBB Model Constructed with bEnd.3 Cells

The integrity of the BBB monolayer model was evaluated based on permeability over 4 h, trans endothelial electrical resistance (TEER), and sodium fluorescein permeation. The initial liquid level difference between the inner and outer chambers of the Transwell inserts was maintained at >0.5 cm. After 4 h, this difference in the control group fell below 0.5 cm, whereas in the cell seeded group it remained above 0.5 cm (Figure 5A). TEER of the bEnd.3 monolayer increased progressively over time and stabilized between days 7 and 9. On day 9, the TEER reached 102.29 Ω·cm^2^, exceeding the standard threshold for an intact barrier [27] (Figure 5B). A calibration curve showed a strong linear correlation between fluorescence intensity and sodium fluorescein concentration, enabling quantitative determination of both concentration and apparent permeability coefficient (Papp). After 120 min, the sodium fluorescein concentration in the lower chamber was 0.639 ± 0.020 µg/mL, corresponding to a Papp of 4.76 × 10^−6^ cm/s, consistent with previously reported values (Figure 5B). Together, these results demonstrate that the bEnd.3-based BBB model possesses complete barrier function, confirming its successful establishment in vitro.

bEnd.3 cells were treated with increasing concentrations of D galactose (0.025 M, 0.05 M, 0.1 M, 0.2 M, 0.4 M) for 24 h. D galactose exposure resulted in a concentration dependent decrease in cell viability, with a reduction of 63% observed at 0.2 M compared to the control (*p* < 0.001) (Figure 5D). Therefore, 0.2 M D galactose was used to establish the injury model in subsequent experiments. Next, cells were treated with different concentrations of PLS (5, 10, 20, 50, 100 μg/mL) following D galactose insult. PLS at 20 μg/mL significantly restored cell viability by 70% (*p* < 0.001) (Figure 5E) and was selected for further BBB permeability assessment.

For the permeability assay, the upper chamber was loaded with 0.5 mL of complete medium containing 20 μg/mL PLS, and the lower chamber with 1.5 mL, ensuring equal liquid levels between compartments. After 4 h of incubation, PLS was detected in the lower chamber across all experimental groups. Although no significant differences in PLS concentration were observed among groups, the presence of PLS in the lower chamber confirmed its ability to cross the in vitro BBB model. Notably, PLS permeability appeared to be enhanced in barriers pre-treated with D galactose, suggesting that D galactose exposure compromises barrier integrity (Figure 5F).

### 3.6. PE-PLS 18:0/20:4 Was Identified as the Principal Bioactive Constituent Responsible for Ameliorating Cognitive Impairments

The two major PLS species detected in the administered preparation, PE-PLS 18:0/20:4 and PE-PLS 18:0/22:6, were also among the predominant species found in the hippocampus. Together with the in vitro BBB permeability results, this finding prompted us to examine their potential interactions with BDNF by molecular docking. 4-Methylcatechol (4-MC) was used as a positive control and showed a predicted binding energy of −6.7 kcal/mol, with interactions involving ARG-87 and TYR-52 (Figure 6A). PE-PLS 18:0/20:4 showed a docking score of −8.3 kcal/mol and formed five hydrogen bonds with THR-81, GLN-83, SER-108, CYS-109, and SER-15 (Figure 6B). PE-PLS 18:0/22:6 showed a docking score of −7.0 kcal/mol and formed hydrogen bonds with CYS-13, CYS-109, SER-15, SER-108, SER-82, SER-17, and TYR-54 (Figure 6C). SER-15, SER-108, and CYS-109 were shared between the two binding modes, indicating that the two PLS species interact with a partly overlapping region of BDNF. However, their overall binding orientations differed. This difference may be related to the sn-2 fatty acyl chain, as PE-PLS 18:0/20:4 and PE-PLS 18:0/22:6 differ in both chain length and degree of unsaturation. These structural differences can affect the conformation and spatial fit of the lipid within the binding region. In addition, although PE-PLS 18:0/22:6 formed more hydrogen bonds, its docking score was less favorable than that of PE-PLS 18:0/20:4, showing that the predicted interaction is not determined by hydrogen bond number alone. Other factors, including hydrophobic contacts, steric fit, and the overall binding conformation, are also likely to contribute.

HT-22 cells were exposed to increasing concentrations of D galactose (0.025 M, 0.05 M, 0.1 M, 0.2 M, 0.4 M) for 24 h. D galactose treatment resulted in a concentration dependent decrease in cell viability, with a reduction of 59% observed at 0.2 M compared with the control (*p* < 0.001) (Figure 6D). Therefore, 0.2 M D galactose was selected to establish the injury model in subsequent experiments. Cells were then treated with different concentrations of PE-PLS 18:0/20:4 (5, 10, 20, and 50 μg/mL) following D galactose insult. Treatment with 20 μg/mL PE Pls 18:0/20:4 significantly restored cell viability by 64.52% (*p* < 0.001) (Figure 6E) and was chosen for further investigation.

To experimentally validate the interaction between PE-PLS 18:0/20:4 and BDNF, a cellular thermal shift assay (CETSA) was performed. At 52 °C, BDNF was significantly degraded in the DMSO-treated group, whereas substantial BDNF levels remained detectable after PLS treatment, indicating that PLS enhances the thermal stability of BDNF (Figure 6F). Furthermore, a rightward shift in the melting curve provided additional support for the stabilizing interaction between PE-PLS 18:0/20:4 and BDNF (Figure 6G). Based on the binding evidence from CETSA, we further evaluated the functional consequences of PE-PLS 18:0/20:4 on BDNF signaling. Treatment with PE-PLS 18:0/20:4 led to functional activation of the BDNF/TrkB pathway and increased the expression of the synaptic markers SYP, PSD-95, and TrkB (Figure 6H–L).

## 4. Discussion

Aging represents the most significant risk factor for cognitive impairment [28]. Cognitive dysfunction associated with brain aging substantially diminishes the quality of life in the elderly, yet effective pharmacological interventions are still lacking [3,28]. In this study, PLS intervention significantly ameliorated cognitive deficits in D-galactose-induced aging mice, confirming its efficacy at the behavioral level (Figure 2B,C). Neurons, as the fundamental units of brain function, are indispensable for information processing and transmission [29,30]. Within the hippocampus, the dentate gyrus (DG) supports neurogenesis and memory formation, the CA3 region is involved in memory storage and spatial navigation, and CA1 contributes to memory integration and consolidation [31,32]. Following D galactose-induced injury, PLS preferentially reduced neuronal degeneration in the hippocampal DG and CA3 subfields, with only modest effects observed in cortical regions (Figure 1D–I). This region specific neuroprotection corresponds to the established vulnerability of hippocampal circuits in age-related cognitive decline and directed our subsequent mechanistic investigations toward hippocampal tissue.

Synapses are essential for neuronal signal transmission and constitute the structural basis of neural circuit function [11]. Both structural and functional synaptic impairments are closely associated with cognitive deficits [13]. PLS treatment alleviated structural synaptic impairments in the hippocampus (Figure 2A–E). Neurotransmitters are stored in presynaptic vesicles and released into the synaptic cleft upon membrane depolarization, where they bind to postsynaptic receptors to relay signals. Thus, neurotransmitters and their receptors play critical roles in synaptic plasticity [27,33]. PLS intervention effectively reversed the abnormal changes in hippocampal levels of 5 HT, NE, and GABA (Figure 3J–L). This finding differs from some earlier reports, a discrepancy that may be attributed to differences in mouse strains or tissue sampling protocols [34]. Furthermore, PLS attenuated the pathological decline in the expression of key synaptic functional proteins, including GluN2A, AMPAR, and GABAR, in the hippocampus (Figure 3A–D). These results align with previously described synaptic protective roles of PLS in various disease and injury models [23,25,26].

The metabolic stability of the vinyl ether bond in PLS during digestion has been a topic of interest. Although vinyl ether bonds are acid-sensitive, no degradation of PLS was observed under simulated gastric and intestinal conditions using a diet containing 10% bovine brain phospholipids. Isotope-labeling studies further confirmed that the vinyl ether bond remains intact during absorption [35], indicating that dietary PLS is likely absorbed in its native form. However, whether PLS can cross the blood–brain barrier (BBB) following gastrointestinal digestion remained unclear. In the present study, oral administration of PLS increased plasmalogen levels in both serum and hippocampus of mice (Figure 4A–D). In parallel, PLS successfully traversed the bEnd.3-based BBB model in vitro (Figure 5A–F). Together, these findings bridge a critical pharmacokinetic gap by demonstrating that orally administered PLS survives digestion and completes the gut–brain transit. This establishes PLS as a credible central nervous system targeting nutrient capable of exerting direct neuroprotective effects.

These findings demonstrate that PLS crosses the BBB and delivers neuroprotective effects. BDNF, a well-established regulator of synaptic plasticity, plays a central role in supporting learning and memory [27]. In cognitively impaired mice, stereotaxic suppression of BDNF expression significantly attenuated the cognitive improvement induced by oral PLS, highlighting BDNF as a potential therapeutic target [34]. However, the mechanism by which exogenous PLS engages BDNF signaling has remained elusive. Takekoshi et al. reported that hippocampal BDNF–TrkB–CREB signaling was enhanced by the combined administration of Chlorella and ascidian-derived PlsEtn, whereas either treatment alone produced no clear activation under their experimental conditions [36]. In contrast, scallop-derived PLS alone modulated BDNF/TrkB-related signaling in the present study, and PE-PLS 18:0/20:4 was further identified as a potential active molecular species interacting with BDNF. These differences may reflect variations in PLS molecular composition or the contribution of antioxidant and other bioactive constituents in Chlorella, and further studies are needed to determine whether BDNF-related signaling represents a general effect of marine PLS or depends on specific PLS molecular species. Our study suggests that PE-PLS 18:0/20:4 exhibits high binding affinity to a defined pocket on BDNF, enhancing its thermal stability. In vitro treatment with PE-PLS 18:0/20:4 was associated with activation of the BDNF/TrkB pathway and upregulation of synaptic proteins SYP and PSD-95, which in turn enhances synaptic plasticity and neuroprotection (Figure 6A–I). Based on these observations, we propose a working hypothesis: during the temporal window between BDNF secretion and TrkB engagement, PLS may “anchor” and “stabilize” BDNF, potentially modulating its local concentration, conformational integrity, and binding kinetics with TrkB—a mechanism that merits further experimental validation. Compositional analysis of the orally administered PLS preparation confirmed that scallop-derived PLS is enriched with PUFAs at the sn-2 position, consistent with previous reports (Figure 6D) [35]. PUFAs have been shown to bind specifically to the N terminal domain of BDNF, possibly inducing conformational changes that improve structural compatibility with TrkB via allosteric effects [37]. Notably, phospholipid bound PUFAs cross the BBB approximately 10-fold more efficiently than their free forms. Collectively, these insights suggest that both the vinyl ether bond and the associated PUFA moiety contribute to the specific and functionally relevant interaction between PLS and BDNF.

## 5. Conclusions

In summary, oral PLS resisted gastrointestinal digestion, entered the bloodstream, crossed the BBB, and accumulated in the hippocampus. PE-PLS 18:0/20:4 emerged as a promising bioactive candidate that may interact with BDNF, potentially contributing to activation of the BDNF/TrkB pathway and improved synaptic plasticity in cognitively impaired mice (Figure 7). Future studies will investigate the downstream signaling pathways of the BDNF/TrkB axis to elucidate the detailed mechanisms underlying PLS-mediated neuroprotection. In vivo comparative studies using purified PE-PLS 18:0/20:4 and other major PLS molecular species will also be conducted to further determine whether PE-PLS 18:0/20:4 represents a principal active component of scallop-derived PLS. In addition, comprehensive combinatorial analyses of multiple active PLS components will be performed to validate the multi-component, multi-target therapeutic potential of scallop-derived PLS extracts.

## Figures and Tables

**Figure 1:**
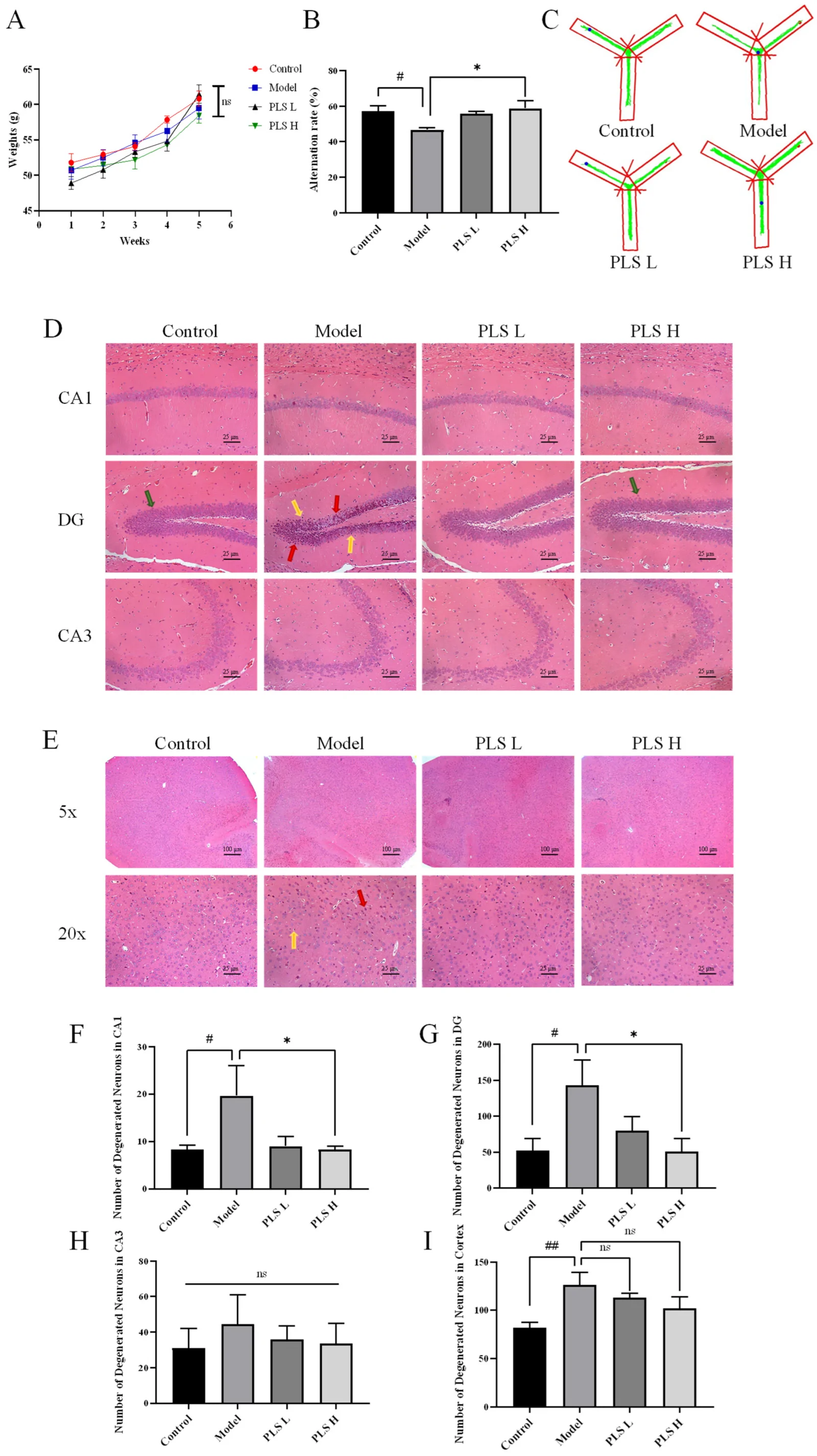
PLS ameliorated memory deficits and neuronal damage in mice: (**A**) Body weight of mice during the intervention period (*n* = 6); (**B**) Alternation rate in the Y-maze test (*n* = 6); (**C**) Representative movement trajectories of mice in the Y-maze; (**D**) Representative micrographs of hippocampal tissue stained with H&E (*n* = 3, 20×), Arrows indicate neurons with distinct morphological features: green, normal neurons; red, shrunken and pyknotic neurons (neurodegeneration); yellow, vacuolated neurons; (**E**) Representative micrographs of cortical tissue stained with H&E (top: 5×, bottom: 20×); (**F**–**I**) Number of degenerated neurons in the (**F**) CA1 region, (**G**) DG region, (**H**) CA3 region and (**I**) the Cortex. Data are presented as mean ± SEM. Statistical significance between groups is represented by # (control vs. model, *p* < 0.05), ## (control vs. model, *p* < 0.001), * (PLS vs. model, *p* < 0.05), ns indicates no significant difference.

**Figure 2:**
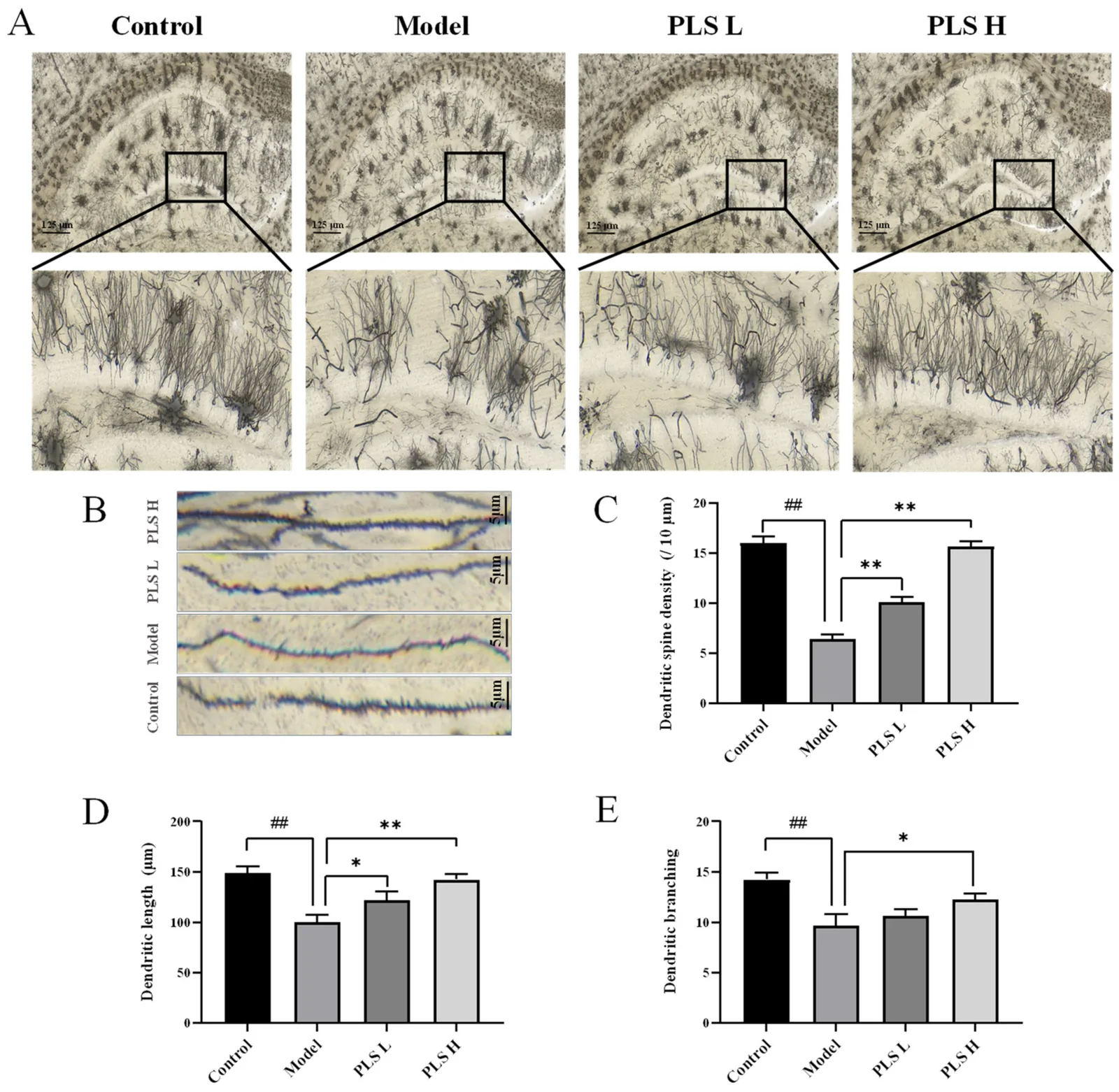
PLS ameliorated synaptic structural impairment in mice with cognitive deficits: (**A**) Representative micrographs of synaptic structures in hippocampal neurons stained by Golgi method (scale bar: 125 μm); (**B**) Representative micrographs of dendritic spines in hippocampal neurons stained by Golgi method (scale bar: 5 μm); (**C**) Dendritic spine density quantified by Image J software (spines per 10 μm); (**D**) Dendritic length of hippocampal neurons quantified by Image J software (μm); (**E**) Dendritic branching of hippocampal neurons quantified by Image J software. Data are presented as mean ± SEM (*n* = 3). Statistical significance between groups is represented by ## (control vs. model, *p* < 0.001), * (PLS vs. model, *p* < 0.05), ** (PLS vs. model, *p* < 0.001).

**Figure 3:**
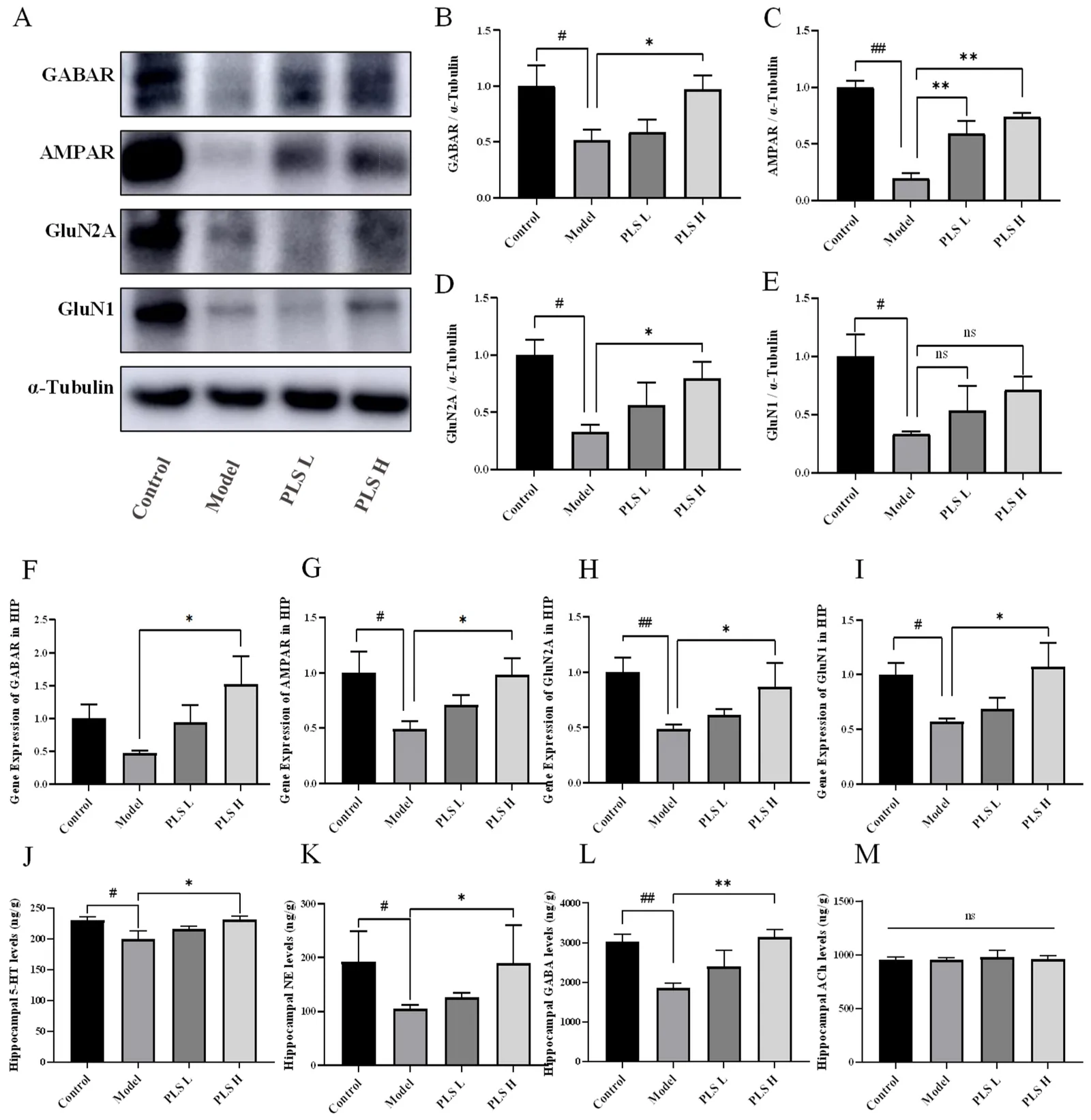
PLS modulated the expression of key synaptic proteins and neurotransmitters: (**A**) Representative Western blot bands of GluN1, GluN2A, AMPAR, and GABAR proteins; (**B**–**E**) Grayscale value analysis of protein bands for (**B**) GABAR, (**C**) AMPAR, (**D**) GluN2A, and (**E**) GluN1 by Image J software (*n* = 3); (**F**–**I**) mRNA analysis of (**F**) GABAR, (**G**) AMPAR, (**H**) GluN2A, (**I**) GluN1 (*n* = 3); (**J**–**M**) Hippocampal 5-HT, NE, GABA and Ach levels measured by ELISA (*n* = 4). Data are presented as mean ± SEM. Statistical significance between groups is represented by # (control vs. model, *p* < 0.05), ## (control vs. model, *p* < 0.001), * (PLS vs. model, *p* < 0.05), ** (PLS vs. model, *p* < 0.001); ns indicates no significant difference.

**Figure 4:**
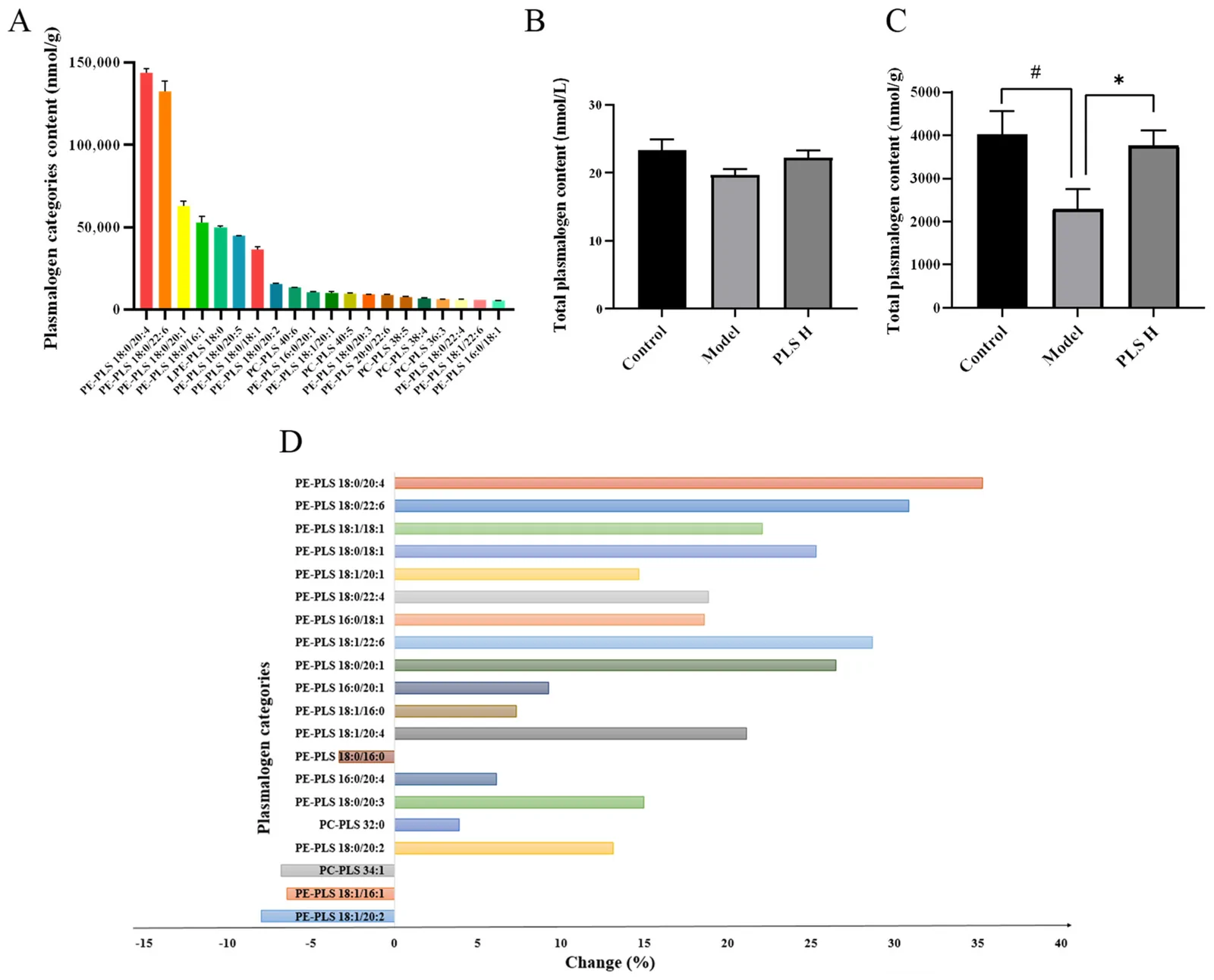
PLS was absorbed and accumulated in the serum and hippocampus after intervention: (**A**) Composition analysis of the orally administered PLS, including PE-PLS (ethanolamine plasmalogen), LPE-PLS (lysophosphatidylethanolamine with plasmalogen), and PC-PLS (choline plasmalogen) (*n* = 3); (**B**,**C**) Total plasmalogen content in (**B**) serum (*n* = 2) and (**C**) hippocampal tissue (*n* = 4); (**D**) Changes in various plasmalogen categories in hippocampal tissue (left bars: downregulated PLS; right bars: upregulated PLS) (*n* = 4). Data are presented as mean ± SEM. Statistical significance between groups is represented by # (control vs. model, *p* < 0.05), * (PLS vs. model, *p* < 0.05).

**Figure 5:**
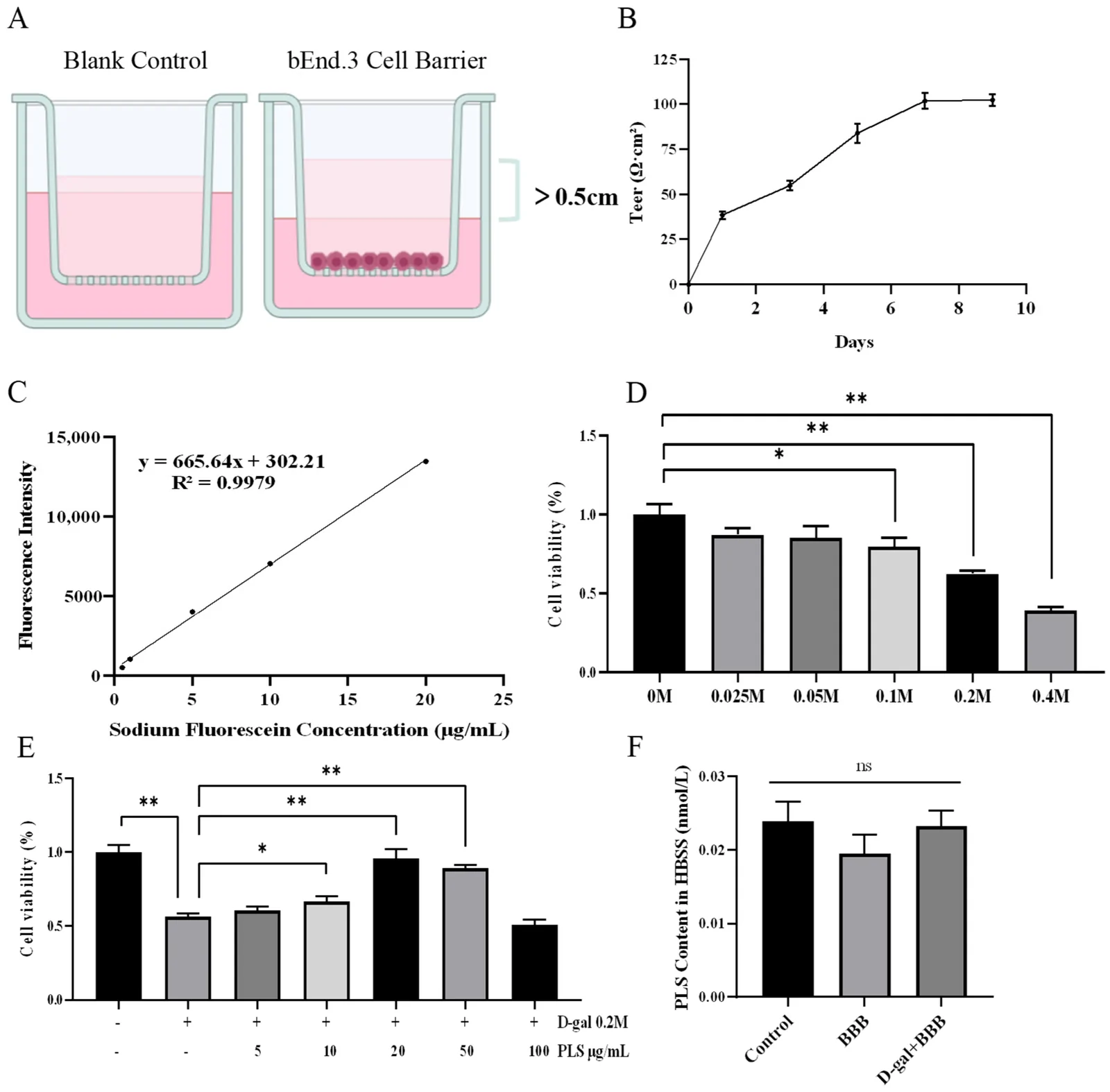
PLS crosses the bEnd.3 cellular blood–brain barrier and specifically binds to BDNF: (**A**) Schematic diagram of liquid level changes in the 4 h permeability assay (left: blank well; right: cell well; *n* = 3); (**B**) Transendothelial electrical resistance (TEER) of the bEnd.3 cell BBB model (*n* = 6); (**C**) Standard curve of sodium fluorescein (*n* = 3); (**D**) Viability of bEnd.3 cells treated with different concentrations of D-gal (*n* = 7); (**E**) Viability of bEnd.3 cells treated with different concentrations of PLS (*n* = 7); (**F**) Content of PE-PLS 18:0/20:4 in the outer chamber of the bEnd.3 BBB model (*n* = 3); data are presented as mean ± SEM. Statistical significance between groups is represented by * (*p* < 0.05), ** (*p* < 0.001); ns indicates no significant difference.

**Figure 6:**
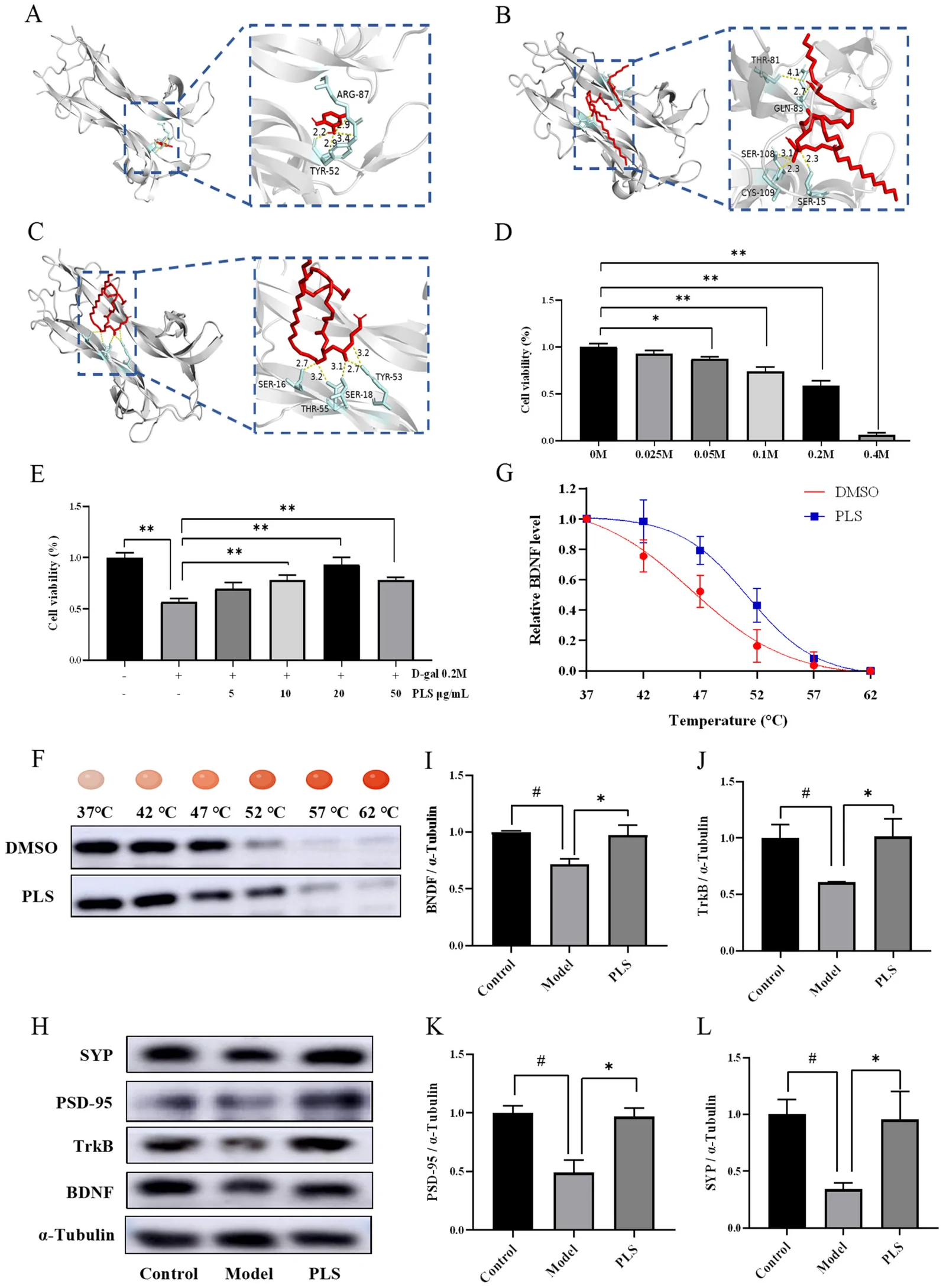
PLS interacts with BDNF and activates the BDNF/TrkB signaling pathway to ameliorate synaptic plasticity: (**A**) Molecular docking simulation of the interaction between 4-MC (positive control) and BDNF; (**B**) Molecular docking simulation of the interaction between PE-PLS 18:0/20:4 and BDNF. (**C**) Molecular docking simulation of the interaction PE-PLS 18:0/22:6 and BDNF. (**D**) Viability of HT-22 cells treated with different concentrations of D-gal (*n* = 7); (**E**) Viability of HT-22 cells treated with different concentrations of PLS (*n* = 7); (**F**) BDNF expression after heat treatment detected by CETSA (*n* = 3); (**G**) Thermal stability curve of BDNF; (**H**) Representative Western blot bands of BDNF, TrkB, PSD-95, and SYP proteins (*n* = 3); (**I**–**L**) Grayscale value analysis of protein bands for (**F**) BDNF, (**G**) TrkB, (**H**) PSD-95, and (**I**) SYP by Image J software. Data are presented as mean ± SEM. Statistical significance is represented by # (control vs. model, *p* < 0.05), * (PLS vs. model or as indicated, *p* < 0.05), ** (*p* < 0.001).

**Figure 7:**
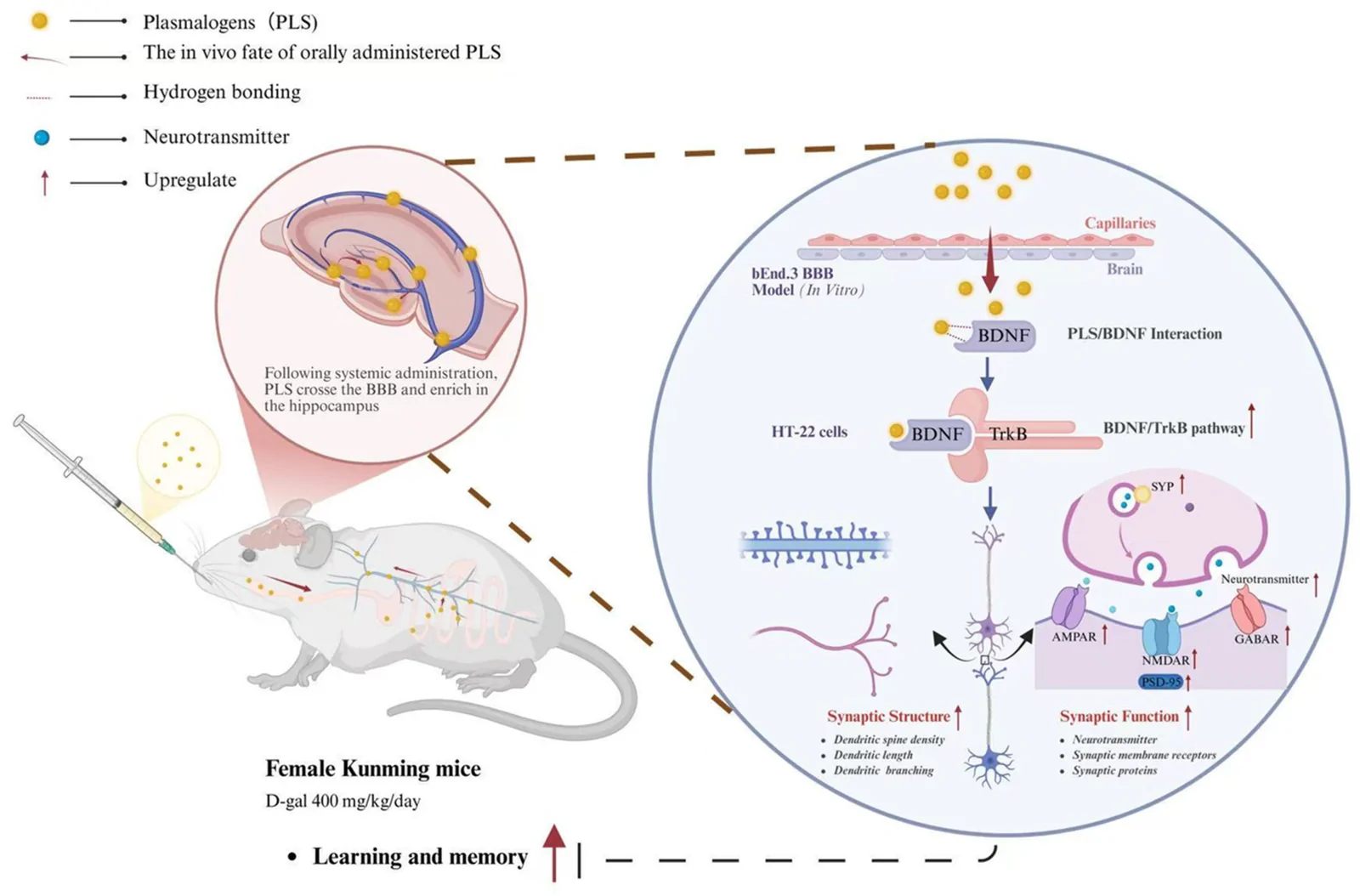
Full-text mechanism diagram: Oral administration of PLS can cross the blood–brain barrier to enter the brain, activate the BDNF/TrkB pathway, enhance synaptic function, and thereby improve cognitive impairment.

## Data Availability

The data presented in this study are available in the article.

## Supplementary Materials

The following supporting information can be downloaded at: https://www.mdpi.com/article/10.3390/nu18193179/s1, Figure S1: Representative MRM chromatograms for the absolute quantification of PE-PLS 18:0/20:4; Table S1: MRM Parameters for Absolute Quantification of PE-PLS 18:0/20:4; Table S2: Information of Authentic Standard and Calibration Curve; Table S3: QC Reproducibility of Normalized Peak Areas for PLS Species in Hippocampal Tissue.

## Abbreviations

The following abbreviations are used in this manuscript:

ACh	Acetylcholine
AMPAR	α-amino-3-hydroxy-5-methyl-4-isoxazolepropionic acid receptor
BBB	Blood–brain barrier
BDNF	Brain-derived neurotrophic factor
CA1	Cornu ammonis area 1
CA3	Cornu ammonis area 3
CETSA	Cellular thermal shift assays
DG	Dentate gyrus
D-gal	D-galactose
GABA	γ-aminobutyric acid
GABAR	γ-aminobutyric acid receptor
MDA	Malondialdehyde
NE	Norepinephrine
GluN1	N-methyl-D- aspartate receptor subunit 1
GluN2A	N-methyl-D-aspartate receptor subunit 2A
Papp	Apparent permeability coefficient
PC-PLS	Choline-type plasmalogens
PE-PLS	Ethanolamine-type plasmalogens
PSD-95	Postsynaptic density protein-95
SOD	Superoxide dismutase
SYP	Synaptophysin
TEER	Transepithelial electrical resistance
TrkB	Tropomyosin receptor kinase B
UPLC-MS/MS	Ultra performance liquid chromatography-tandem mass spectrometry
5-HT	5-Hydroxytryptamine

## Appendix A

**Table A1:** Instrumental Parameters and Method Settings for UPLC-MS/MS Analysis.

Parameter	Setting/Specification
Column	Waters ACQUITY UPLC BEH C18 (2.1 mm × 100 mm, 1.7 μm particle size)
Column Temperature (°C)	40
Autosampler Temperature (°C)	10
Mobile Phase	A: Acetonitrile/water (60:40, *v*/*v*) with 5 mM ammonium acetate and 0.1% (*v*/*v*) formic acid B: Isopropanol/acetonitrile (90:10, *v*/*v*) with 5 mM ammonium acetate and 0.1% (*v*/*v*) formic acid.
Gradient Program (Time, min/%B)	0.0/60, 0.5/60, 11.0/100, 13.0/100, 13.5/60, 15.0/60
Flow Rate (mL min^−1^)	0.30
Injection Volume (μL)	2.0
Ionization Source	Electrospray ionization (ESI), positive mode
Capillary Voltage (kV)	3.0
Source Temperature (°C)	150
Desolvation Temperature (°C)	550
Desolvation Gas Flow (L h^−1^)	1000
Collision Gas Flow (L h^−1^)	0.13
