# Supplementary material for: Blood–Brain Barrier (BBB)-Permeable Ethanolamine-Type Plasmalogens 18:0/20:4 (PE-PLS 18:0/20:4) Ameliorate Cognitive Impairments in Aged Mice
Source: Nutrients. 2026 Sep 26;18(19):3179. doi: 10.3390/nu18193179 (PMC13666644; doi:10.3390/nu18193179)
Supplement: Supplementary file 1 [file nutrients-18-03179-s001.zip › Supplementary Figure S1.pdf]

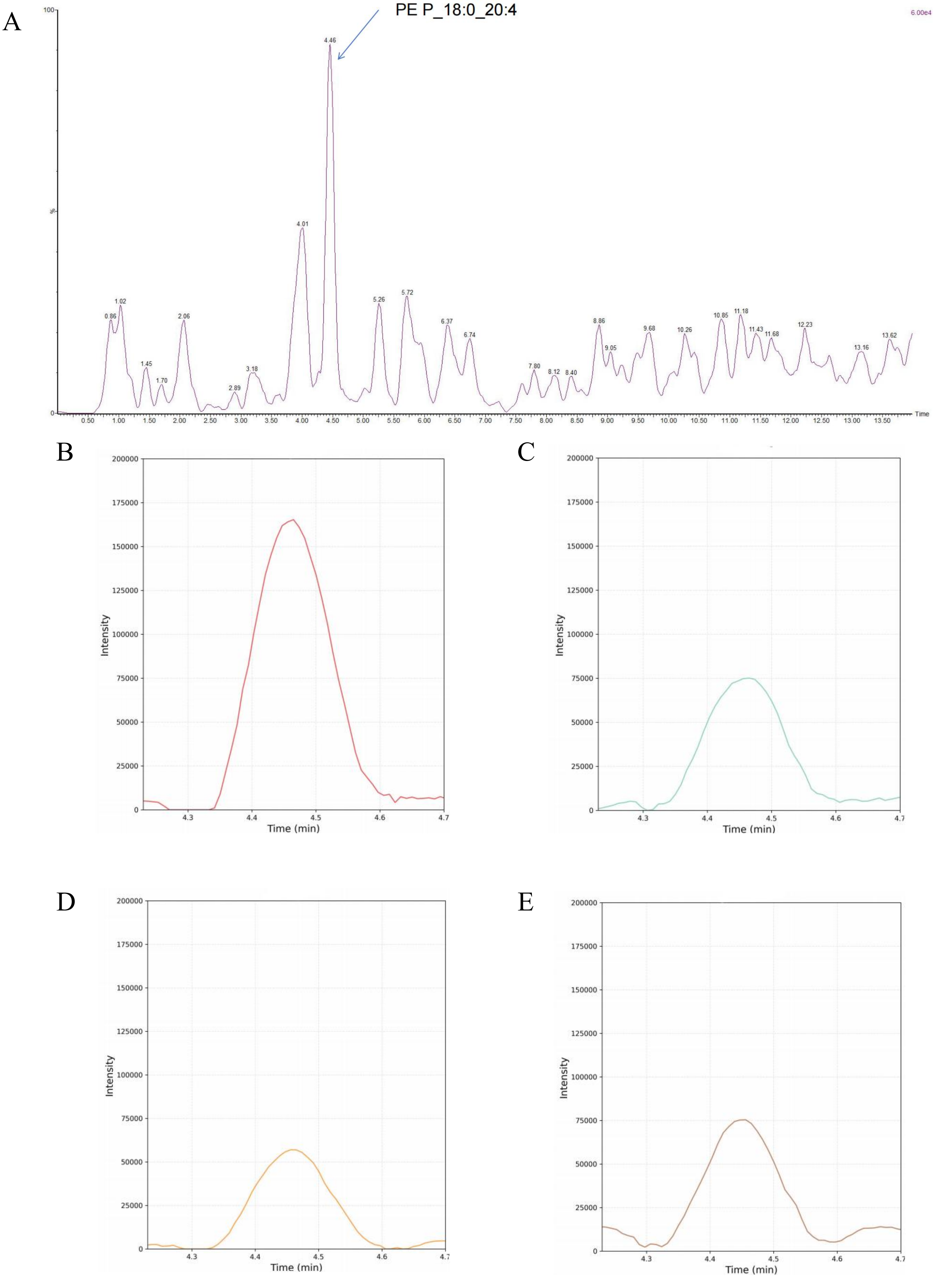

Supplementary Figure S1：
Representative MRM chromatograms for the
absolute quantification of PE-PLS 18:0/20:4. (A) QC sample.
(B) Authentic standard of PE-PLS 18:0/20:4.(C–E) Representative
samples from the three experimental groups: (C) Control, (D) BBB,
and (E) D-gal+BBB.
