# Supplementary material for: Blood–Brain Barrier (BBB)-Permeable Ethanolamine-Type Plasmalogens 18:0/20:4 (PE-PLS 18:0/20:4) Ameliorate Cognitive Impairments in Aged Mice
Source: Nutrients. 2026 Sep 26;18(19):3179. doi: 10.3390/nu18193179 (PMC13666644; doi:10.3390/nu18193179)
Supplement: Supplementary file 1 [file nutrients-18-03179-s001.zip › Supplementary Tables S1-S3.pdf]

Supplementary Tables S1: MRM Parameters for Absolute Quantification of PE-PLS 18:0/20:4.

Parameter	Value
Ion mode	ESI+
Precursor ion (Q1, m/z)	752
Product ion (Q3, m/z)	361
Collision energy (eV)	17 (literature value)

Table note: The MRM transition for PE-PLS 18:0/20:4 was 752 > 361 in positive ion mode (ESI+). The collision energy is based on the value reported in the Baker Metabolomics Portal and published literature for the same MRM transition, and will be updated upon confirmation from the analytical laboratory.

Supplementary Tables S2: Information of Authentic Standard and Calibration Curve.

Parameter	Value
Compound name	PE-PLS 18:0/20:4
Lipid class	PE plasmalogen
Purity	>99% (TLC)
Manufacturer	Avanti Polar Lipids
Catalog number	852804C
Retention time (min)	4.46 (QC sample)
Calibration curve	$y = 1020.0x$, $R^2 = 0.9996$

Supplementary Tables S3: QC Reproducibility of Normalized Peak Areas for PLS Species in Hippocampal Tissue.

Species	QC1	QC2	RPD (%)
PE-PLS 18:1/20:2	9.6907	10.1570	4.70
PE-PLS 18:1/16:1	10.1162	10.2411	1.23
PC-PLS 34:1	11.5522	10.9583	5.28
PE-PLS 18:0/20:2	10.0244	10.1192	0.94
PC-PLS 32:0	16.3344	15.7760	3.48
PE-PLS 18:0/20:3	18.8004	18.2029	3.23
PE-PLS 16:0/20:4	21.3330	22.4436	5.07
PE-PLS 18:0/16:0	34.1129	34.0278	0.25
PE-PLS 18:1/20:4	56.6089	56.5604	0.09
PE-PLS 18:1/16:0	104.2945	101.5515	2.67
PE-PLS 16:0/20:1	86.6833	87.7438	1.22
PE-PLS 18:0/20:1	143.0975	137.1854	4.22
PE-PLS 18:1/22:6	208.0486	205.5389	1.21
PE-PLS 16:0/18:1	191.2891	189.1500	1.12
PE-PLS 18:0/22:4	233.4628	235.7868	0.99
PE-PLS 18:1/20:1	245.8221	254.6369	3.52
PE-PLS 18:0/18:1	362.1746	371.2831	2.48
PE-PLS 18:1/18:1	464.6139	498.5561	7.05
PE-PLS 18:0/22:6	678.9189	693.3346	2.10
PE-PLS 18:0/20:4	754.8937	747.0781	1.04

Table note: The number of QC samples was 2, precluding calculation of RSD. RPD = $|QC1 - QC2| / [(QC1 + QC2)/2] \times 100\%$. All PLS species showed RPD < 15%.
